# Effect of fenofibrate in 1113 patients at low-density lipoprotein cholesterol goal but high triglyceride levels: Real-world results and factors associated with triglyceride reduction

**DOI:** 10.1371/journal.pone.0205006

**Published:** 2018-10-04

**Authors:** Yeongmin Woo, Jeong-soo Shin, Chi-Young Shim, Jung-Sun Kim, Byeong-Keuk Kim, Sungha Park, Hyuk-Jae Chang, Geu-Ru Hong, Young-Guk Ko, Seok-Min Kang, Donghoon Choi, Jong-Won Ha, Myeong-Ki Hong, Yangsoo Jang, Sang-Hak Lee

**Affiliations:** 1 Division of Cardiology, Department of Internal Medicine, Gangneung Asan Hospital, University of Ulsan College of Medicine, Gangneung, Korea; 2 Department of Biostatistics and Computing, Yonsei University College of Medicine, Seoul, Korea; 3 Division of Cardiology, Department of Internal Medicine, Severance Hospital, Yonsei University College of Medicine, Seoul, Korea; 4 Cardiovascular Research Institute, Yonsei University College of Medicine, Seoul, Korea; University of Milano, ITALY

## Abstract

Fibrates are used in patients with dyslipidemia and high cardiovascular risk. However, information regarding drug response to fibrate has been highly limited. We investigated treatment results and factors associated with triglyceride reduction after fenofibrate therapy using large-scale real-world data. Patients with one or more cardiovascular risk factors, at low-density lipoprotein-cholesterol goal but with triglyceride level ≥150 mg/dL, and undergoing treatment with fenofibrate 135–160 mg for the first time were included in this retrospective observational study. The outcome variable was the percentage changes of TG levels. The achievement rate of triglyceride <150 mg/dL was additionally analyzed. Factors associated with treatment results were also analyzed. Among 2546 patients who were initially screened, 1113 patients were enrolled (median age: 61 years; male: 71%). After median follow-up of 4 months, the median change in triglyceride was -60%, and 49% of the patients reached triglyceride <150 mg/dL. After adjusting for confounding variables, female sex, non-diabetic status, coronary artery disease, lower baseline triglyceride, and no statin use were identified to be independently associated with achievement of triglyceride <150 mg/dL. Among them, female sex, non-diabetic status, and coronary artery disease were also related to median or greater percentage reduction of triglyceride. In conclusion, only half of the study patients reached triglyceride levels <150 mg/dL after real-world fenofibrate therapy. This study indicates that more attention is needed on some subgroups to obtain optimal triglyceride levels when treating with fenofibrate.

## Introduction

Fibrate is frequently used in patients with dyslipidemia and cardiovascular risk [1–3]. Its clinical benefit has been shown in specific conditions such as atherogenic dyslipidemia [4–7] or diabetes for retardation of microangiopathy [8, 9]. However, universal control of triglyceride (TG) is not strongly recommended for cardiovascular prevention [3, 10] and real-world data regarding drug response to fibrate has been highly limited.

Fibrate therapy has been known to change TG levels by around -40% in clinical trials [11–14]. However, the efficacy of lipid-lowering agents can be varied to some degree according to individual’s characteristics [15]. Several papers have reported genetic determinants of fenofibrate response [16–19]. In addition, there are some studies that evaluated variables associated with response to the medication such as age and cardiovascular risk factors [20]. However, analysis on such determinants has been insufficient and the results were inconsistent [15, 21–23], particularly for fibrates.

Dyslipidemia pattern has been reported diverse according to different ethnicities. In a large previous analysis, the majority of patients with cardiovascular disease were reported to have history of hypertriglyceridemia. It is remarkable that Koreans (particularly men) showed higher prevalence of hypertriglyceridemia [24] than other ethnicities. Proper evaluation of treatment results and management of dyslipidemia in a population with high prevalence of this condition is important. Furthermore, identifying the variables associated with efficacy of drugs affecting TG levels in such population may be helpful. The aim of the current study was to investigate the results of treatment using the usual dose of fenofibrate in real-world practice. We also analyzed data to identify the variables associated with reduction of TG levels.

## Materials and methods

### Study population

The Institutional Review Board of Severance Hospital, Seoul, Korea approved this study. Informed consent was waived because the research involves no more than minimal risk to the subjects, the waiver does not reversely affect the rights and welfare of the subjects, and the research could not be practicably carried out without the waiver. Study subjects were enrolled in the outpatient clinic of the Division of Cardiology, Severance Hospital, Seoul, Korea, from April 2005 to December 2015.

Patients with TG levels >150 mg/dL and low-density lipoprotein-cholesterol (LDL-C) lower than the target levels as defined by NCEP ATP III guidelines [25], with one or more cardiovascular risk factors, and undergoing treatment with fenofibrate for the first time were included. Fenofibrate regimens included fenofibrate 160 mg (Lipidil Supra; Green Cross Corp., Yongin, Korea) and fenofibric acid 135 mg (Fenocid; Hanmi Pharmaaceutical, Seoul, Korea). We excluded patients who were followed up for <3 months, who did not undergo follow-up laboratory examination, and who changed the lipid-modifying regimen within 3–9 months. No patients were newly prescribed with statins during the follow-up period.

### Study protocol

This was retrospective observational study. Clinical information, including demographic variables, medical history, and medication use, were obtained by trained interviewers. Blood samples were collected after 12-h fasting and analyzed by the local laboratory certified by the Korean Society of Laboratory Medicine. Atherogenic dyslipidemia was defined as the presence of ≥TG levels 200 mg/dL and high-density lipoprotein-cholesterol (HDL-C) ≤40 and 50 mg/dL in males and females [26]. The study participants were treated for their medical conditions using standard therapies. The patients were followed every 3–6 months in the outpatient clinic. They underwent blood test for lipid profile every 3–6 months. Post-treatment TG level was defined as the level during examination at 6±3 months after starting treatment. If tests were performed more than once during the period, post-treatment TG level was defined as the average of the levels examined during the period. The outcome variable was percentage changes of TG levels. The achievement rate of TG <150 mg/dL was additionally analyzed. Percentage changes of TG levels were calculated as follows: (post-treatment value–pre-treatment value) / pre-treatment value × 100 (%). The optimal level of TG was defined as <150 mg/dL, which was suggested as desirable in multiple major consensuses on lipid management, particularly in high-risk patients [3, 12, 27]. Data are available in S1 File.

### Statistical analysis

Continuous variables are reported as median (interquartile ranges). Categorical variables are presented as frequencies and percentages. Patients’ clinical and laboratory variables were compared by using Mann-Whitney U test and the chi-square test. Wilcoxon signed rank test was used to compare parameters before and after drug treatment within a group of patients. The variables associated with reduction of TG levels were identified through a univariate regression analysis. Age, sex, and variables with *p*<0.15 in the analysis were entered into a multiple logistic regression analysis in a stepwise manner. Odds ratios and 95% confidence intervals were calculated. All analyses used two-tailed tests with a significance level of 0.05. SPSS version 17.0 (SPSS Inc., Chicago, IL, USA) was used for all analyses.

## Results

### Clinical characteristics

Among 2546 patients who were initially screened, 1433 were excluded, and 1113 patients were finally enrolled to the study (details are described in S1 Fig). Patients’ median age was 61 years and 71% were men. Forty percent of the participants were diabetic, whereas 38% had coronary artery disease (CAD). Thirty-one percent of the patients were concomitantly taking statins. The median pre-treatment TG level was 357 mg/dL (Table 1).

**Table 1:** Clinical and laboratory parameters of the study population.

	Total population (N = 1,113)
Age, years	61 (51, 69)
Male (%)	787 (70.7)
Medical history (%)	
Diabetes mellitus	442 (39.7)
Hypertension	904 (81.2)
Current smoking	238 (21.4)
Coronary artery disease	426 (38.3)
Stroke	65 (5.8)
Body mass index, kg/m^2^	25.2 (23.2, 27.5)
Current statin use	347 (31.2)
Pre-treatment laboratory values, mg/dL	
Total cholesterol	187 (163, 213)
Triglyceride	357 (266, 501)
HDL-C	38.0 (33.0, 43.0)
LDL-C	83 (64, 100)
Glucose	106 (96, 124)
Post-treatment laboratory values, mg/dL	
Total cholesterol	173 (152, 231)*
Triglyceride	152 (111, 231)*
HDL-C	43.0 (38.0, 50.0)*
LDL-C	100 (79, 121)*
Glucose	103 (93, 124)*

Data are presented as median (interquartile range) or n (%). HDL-C: high-density lipoprotein-cholesterol; LDL-C: low-density lipoprotein-cholesterol.

* *p* <0.001, compared to pre-treatment values.

### Changes in lipid parameters

**Fig 1:**
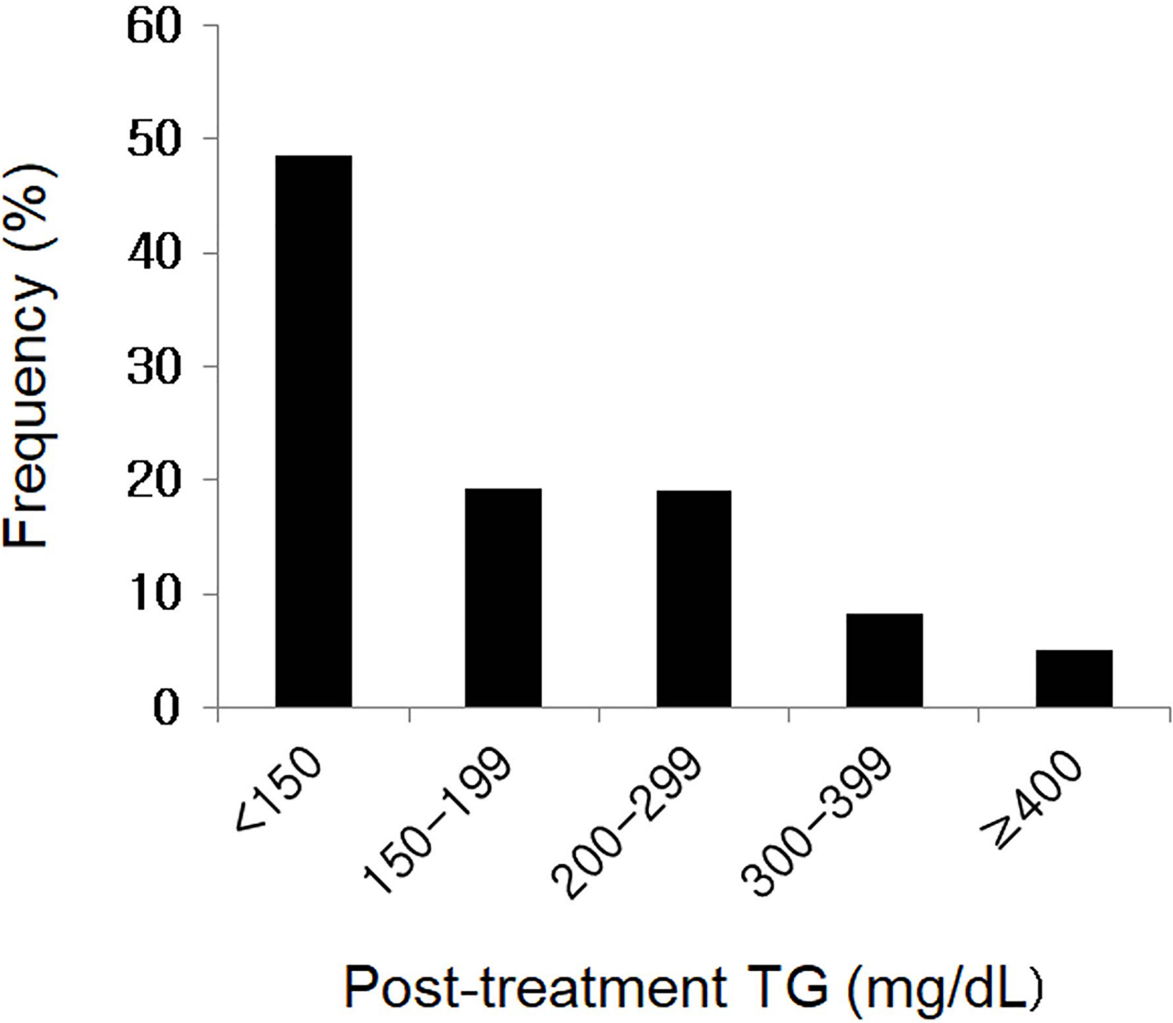
Distribution of post-treatment TG levels in the study population (n = 1153). TG: triglyceride.

The median time of laboratory follow-up was 4 months (range: 2–9 months; interquartile range: 3–6 months). After treatment with fenofibrate, the median changes in total cholesterol, TG, HDL-C, LDL-C, and glucose were -6.5%, -60.0%, 14.3%, 17.3%, and -2.2%, respectively (*p*<0.001 for all) (Table 1). Data regarding optimal level achievement are presented in Fig 1. Forty-nine percent of the participants achieved TG <150 mg/dL, whereas 19%, 19%, and 8% of the patients reached post-treatment TG levels of 150–199, 200–299, and 300–399 mg/dL, respectively (Fig 1).

### Subgroup analysis and variables associated with TG reduction

The percentage reduction of TG was greater in women, patients without diabetes mellitus (DM), non-smokers, those with pre-treatment TG ≥500 mg/dL or median, with atherogenic dyslipidemia, or without statin use (Table 2). Conversely, achievement rate of TG <150 mg/dL was higher in patients with median age or older, females, those without DM, non-smokers, patients with CAD, with body mass index (BMI) lower than median, or with pre-treatment TG <500 mg/dL or lower than median (S2 Fig).

**Table 2:** Subgroup analysis for changes of TG after treatment with fenofibrate.

	Subgroup		P
	Age ≥Median (n = 596)	Age < Median (n = 517)	
Before	340 (250, 461)	394 (290, 577)	<0.001
After	139 (104, 194)	179 (122, 276)	<0.001
% change	-57.8 (-70.9, -39.7)	-56.3 (-69.5, -37.2)	0.32
P	<0.001	<0.001	
	Male (n = 787)	Female (n = 326)	
Before	349 (265, 507)	375 (267, 487)	0.99
After	162 (118, 243)	133 (97, 194)	<0.001
% change	-54.7 (-67.6, -36.1)	-61.0 (-73.8, -47.5)	0.002
P	<0.001	<0.001	
	DM (n = 442)	No DM (n = 671)	
Before	373 (261, 531)	347 (269, 476)	0.42
After	165 (122, 245)	146 (106, 217)	0.001
% change	-52.9 (-68.6, -32.7)	-59.3 (-71.0, -43.3)	0.001
P	<0.001	<0.001	
	Hypertension (n = 904)	No hypertension (n = 209)	
Before	358 (264, 501)	355 (271, 495)	0.20
After	153 (111, 231)	152 (104, 229)	0.51
% change	-56.0 (-69.4, -38.2)	-59.2 (-72.1, -40.3)	0.29
P	<0.001	<0.001	
	Smoking (n = 238)	No smoking (n = 875)	
Before	348 (269, 532)	359 (265, 487)	0.18
After	172 (124, 244)	146 (106, 220)	0.02
% change	-55.0 (-68.6, -34.5)	-57.5 (-70.9, -39.3)	0.02
P	<0.001	<0.001	
	CAD (n = 426)	No CAD (n = 687)	
Before	325 (247, 442)	386 (282, 547)	<0.001
After	136 (99, 205)	160 (119, 243)	<0.001
% change	-57.6 (-70.4, -36.2)	-56.3 (-69.8, -39.9)	0.61
P	<0.001	<0.001	
	BMI ≥Median (n = 431)	BMI <Median (n = 442)	
Before	358 (268, 485)	339 (256, 496)	0.70
After	160 (114, 241)	143 (103, 211)	0.03
% change	-57.4 (-69.1, -36.8)	-57.8 (-70.9, -39.0)	0.92
P	<0.001	<0.001	
	Pre-treatment TG ≥500 mg/dL (n = 282)	Pre-treatment TG <500 mg/dL (n = 831)	
Before	683 (577, 879)	339 (248, 388)	<0.001
After	225 (145, 319)	138 (103, 194)	<0.001
% change	-67.4 (-79.2, -55.0)	-53.2 (-65.8, -34.9)	<0.001
P	<0.001	<0.001	
	Pre-treatment TG ≥Median (n = 558)	Pre-treatment TG <Median (n = 555)	
Before	501 (420, 691)	266 (227, 310)	<0.001
After	194 (129, 281)	131 (97, 175)	<0.001
% change	-63.3 (-74.6, -48.6)	-50.0 (-61.4, -31.7)	<0.001
P	<0.001	<0.001	
	Atherogenic dyslipidemia (n = 756)	No atherogenic dyslipidemia (n = 350)	
Before	378 (280, 524)	308 (229, 468)	<0.001
After	154 (112, 229)	149 (107, 236)	0.29
% change	-58.7 (-71.2, -40.6)	-54.2 (-65.2, -32.2)	<0.001
P	<0.001	<0.001	
	Statin use (n = 351)	No statin use (n = 762)	
Before	312 (247, 428)	392 (275, 534)	<0.001
After	151 (106, 210)	153 (112, 239)	0.11
% change	-54.6 (-67.9, -33.8)	-58.4 (-70.9, -41.1)	0.006
P	<0.001	<0.001	

TG: triglyceride; DM: diabetes mellitus; CAD: coronary artery disease; BMI: body mass index.

After adjusting confounding variables in the multiple logistic regression analysis, old age, female, absence of DM, history of CAD, and higher pre-treatment TG were found to be independently associated with median or greater percentage reduction of TG (Table 3). Variables associated with higher achievement rate of TG <150 mg/dL are shown in S1 Table.

**Table 3:** Variables associated with TG reduction identified by multiple logistic regression analysis.

Variables	≥median % reduction of TG (n = 553)	<median % reduction of TG (n = 553)	OR (95% CI)	P
Age, year	60.7 ± 11.8	60.3 ± 11.7	0.99 (0.98, 1.00)	0.04
Male	351 (63.7)	430 (77.8)	2.12 (1.53, 2.94)	<0.001
Diabetes mellitus	191 (34.5)	246 (44.5)	1.97 (1.48, 2.61)	<0.001
Hypertension	440 (79.6)	459 (83.0)	1.34 (0.95, 1.90)	0.09
Current smoking	110 (19.9)	128 (23.1)	1.00 (0.72, 1.39)	0.99
CAD	218 (39.4)	208 (37.6)	0.53 (0.39, 0.73)	<0.001
Pre-treatment TG	417 (312, 636)	305 (238, 422)	0.996 (0.995, 0.997)	<0.001
Atherogenic dyslipidemia	404 (73.1)	352 (63.7)	0.92 (0.69, 1.23)	0.58
Statin use	157 (28.4)	194 (35.1)	1.18 (0.87, 1.60)	0.28

TG: triglyceride; OR: odds ratio; CI: confidence interval; CAD: coronary artery disease

## Discussion

In the current study, the median reduction of TG by usual dose of fenofibrate was -60%, whereas 49% of patients achieved TG <150 mg/dL. The percentage of TG reduction was greater in women, patients without DM, non-smokers, and those with higher pre-treatment TG, with atherogenic dyslipidemia, or without statin use. After adjusting confounding variables, older age, female sex, non-diabetic status, history of CAD, and higherpre-treatment TG level were identified to be associated with optimal level achievement. Taken together, in real-world practice, only half of patients receiving fenofibrate therapy reached the optimal TG levels and more attention may be needed to subgroups such as males or diabetic patients.

The median change in TG in our study was -60% and is relatively greater than those of prior reports. A few recent studies performed in Asia and the United States showed percentage change ranging from -36% to -43% [11, 13, 14]. Of note, a study performed in our group revealed TG reduction down to -53% [1]. Although the reason for relatively greater reduction in our population is not clear by current data, patients’ characteristics such as higher baseline TG levels and different rate of statin use might have caused the difference.

We analyzed and identified several variables associated with TG reduction in the present study. To date, studies evaluating factors affecting TG control in considerable size of population have been very scarce. For example, age ≥65 years was a negative predictor for non-HDL-C goal attainment in patients with dyslipidemia [21]. Meanwhile, a Taiwanese study showed a positive association between age and achievement rate of LDL-C target [15]. Interestingly, the FIRST trial identified that factors including older age and pre-existing CAD, are associated with reductions in carotid intima media thickness after treatment with fenofibric acid, although the evaluation point was not the same as ours [28]. In our population with median age of 61 years, age showed a positive correlation with ≥median % reduction of TG levels. A prior study indicated that age had relation with drug adherence, but its direction was not consistent throughout the range of age [22]. This finding is in accordance with the association between age and drug efficacy shown in our study.

Our data revealed that TG reduction was greater in women than in men. To our knowledge, our study is the first to evaluate sex difference regarding fenofibrate effect on TG reduction. An analysis of the FIELD study showed that LDL-C or apoB reduction was greater in women [23]. On the contrary, the target achievement rate of non-HDL-C [21] as well as drug adherence [29] was lower in women. Sex difference in body mass or volume of distribution has been suggested as potential causes [23]. In addition, serum concentrations of fenofibric acid have been reported to be 1.3-fold higher in women than in men in a prior study [30]. However, it is difficult to fully understand the mechanism of sex differences in our results at present.

Of note, DM was associated with lower than median percentage reduction of TG. Although studies on the relationship between DM and TG reduction are extremely limited, a prior study has demonstrated that DM was the most important determinant of failure to attain the non-HDL-C goal [21]. Patients with DM frequently exhibit a pattern of high TG and low HDL-C, and people with this atherogenic dyslipidemia are known to benefit from fibrate treatment [31]. In this regard, our current study indicates that more attention to this population is needed, because they often get suboptimal lipid modifying results by fenofibrate treatment. CAD had positive association with TG reduction in the current study. It is known that adherence of patients to lipid-lowering therapy is higher in population of secondary prevention compared with that of primary prevention [15, 22]. This may be related to risk perception about CAD by patients and subsequently improved adherence. In this regard, although data on the adherence were not collected in our study, the better effect of fenofibrate in CAD patients may be partly due to different drug adherence in this subgroup. In addition, pre-treatment TG levels were lower in the CAD group, and this might have affected our results.

Above all, the strength of our study is that it is the first large real-world analysis of the fenofibrate effect on TG in patients with LDL-C at goal. Although there are several reports that handled similar issue as mentioned above, they were smaller and did not systematically analyze factors associated with the drug efficacy. Our study has a few potential limitations. The lack of a control group not receiving treatment is an important limitation of our study. In our attempt to find such patients, only a population that was too small with insufficient clinical data was available. Due to the lack of a control group, we could not derive any conclusion on causality. For example, factors that showed association with TG reduction after treatment can be related to other confounding factors. Although it is difficult to obtain a perfect control group in the real world, it might have allowed us to draw clearer conclusions. The current analysis does not include sufficient data for drug adherence. Because adherence is one of the important factors affecting treatment results, addition of this information might have made our study more complete. In addition, our primary evaluation parameter was percentage TG reduction. In patients with high LDL-C plus high TG levels, non-HDL-C is used as one of treatment targets. However, because we only enrolled patients already at LDL-C goal, evaluation of treatment effect with fenofibrate in these people with TG reduction may be regarded appropriate. Finally, the current study was conducted in Koreans. Therefore, further studies may be needed to show whether our results are applicable to other ethnicities.

Taken together, in real-world practice, the usual dose of fenofibrate reduced TG to <150 mg/dL in only half of patients at LDL-C goal but with hypertriglyceridemia. Several clinical variables including female sex, non-diabetic status, and history of CAD were identified to be independently associated with reduction of TG levels. These results indicate that more attention is needed to subgroups such as males or with diabetes when treating this population with fenofibrate.

## Supporting information

S1 Fig Patient screening, exclusion, and enrollment. (TIF)

S2 Fig Achievement rate of TG <150 mg/dL by fenofibrate in the subgroups. DM: diabetes mellitus; CAD: coronary artery disease; BMI: body mass index.
(TIF)

S1 Table Variables associated with achievement rate of TG <150 mg/dL identified by multiple logistic regression analysis. (DOCX)

S1 File Data. (DOCX)
