# Supplementary figures and images for: Effect of fenofibrate in 1113 patients at low-density lipoprotein cholesterol goal but high triglyceride levels: Real-world results and factors associated with triglyceride reduction
Source: PLoS One. 2018 Oct 4;13(10):e0205006. doi: 10.1371/journal.pone.0205006 (PMC6171908; doi:10.1371/journal.pone.0205006)

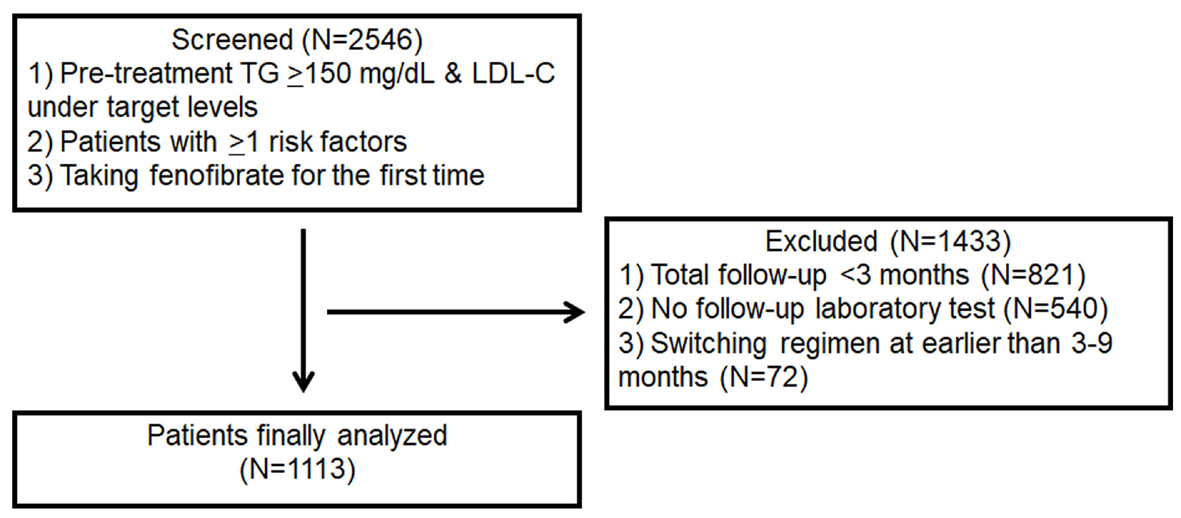

Supplement: S1 Fig — (TIF) [file pone.0205006.s001.tif]

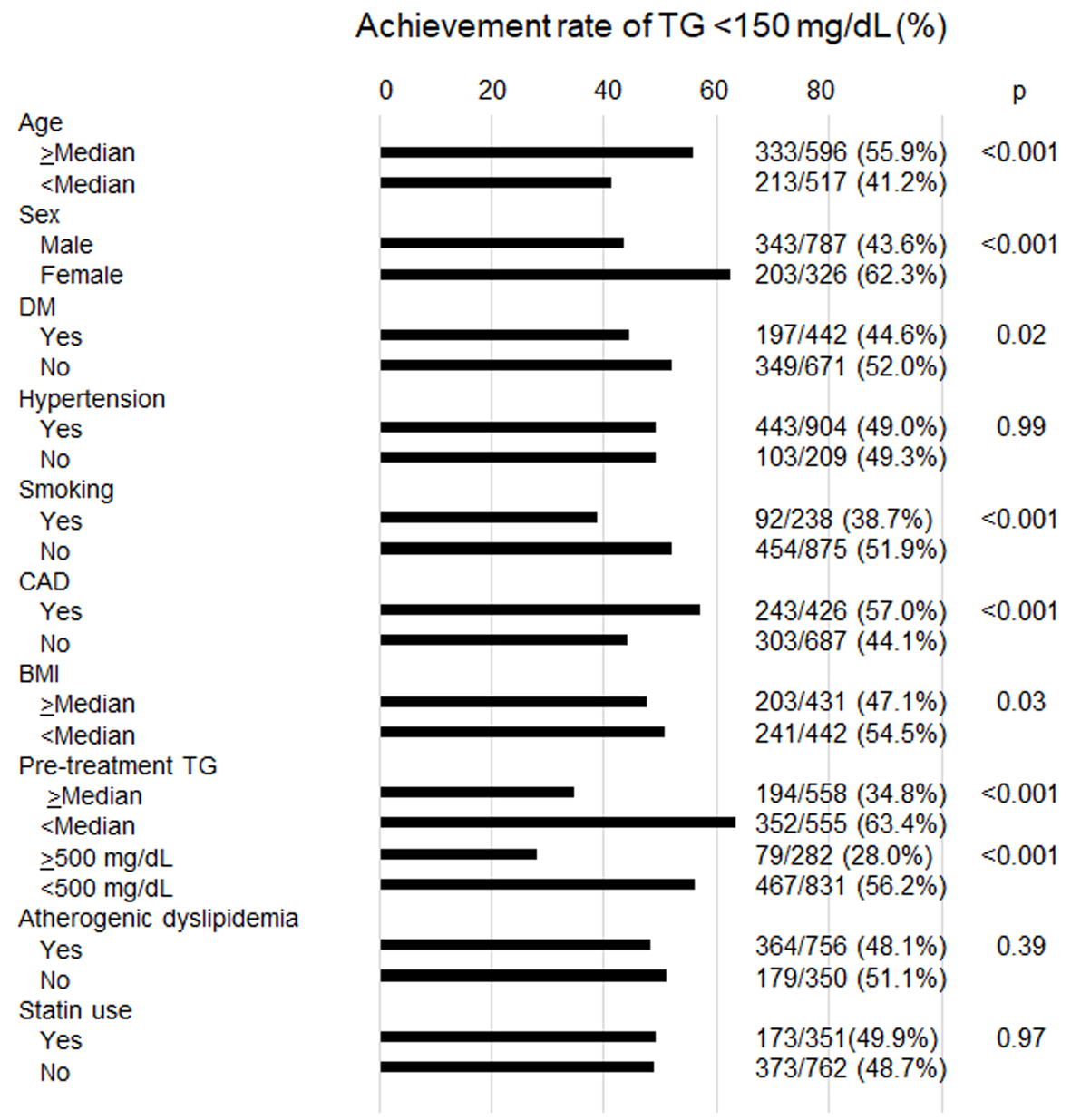

Supplement: S2 Fig — DM: diabetes mellitus; CAD: coronary artery disease; BMI: body mass index. (TIF) [file pone.0205006.s002.tif]
