# Supplementary material for: Effect of fenofibrate in 1113 patients at low-density lipoprotein cholesterol goal but high triglyceride levels: Real-world results and factors associated with triglyceride reduction
Source: PLoS One. 2018 Oct 4;13(10):e0205006. doi: 10.1371/journal.pone.0205006 (PMC6171908; doi:10.1371/journal.pone.0205006)
Supplement: S1 File — (DOCX) [file pone.0205006.s004.docx]

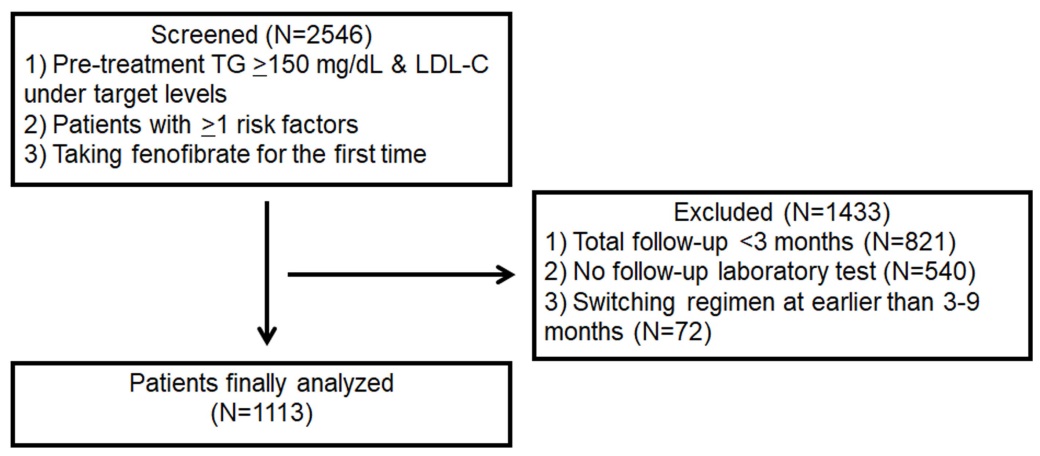


**S1 Fig. Patient screening, exclusion, and enrollment**


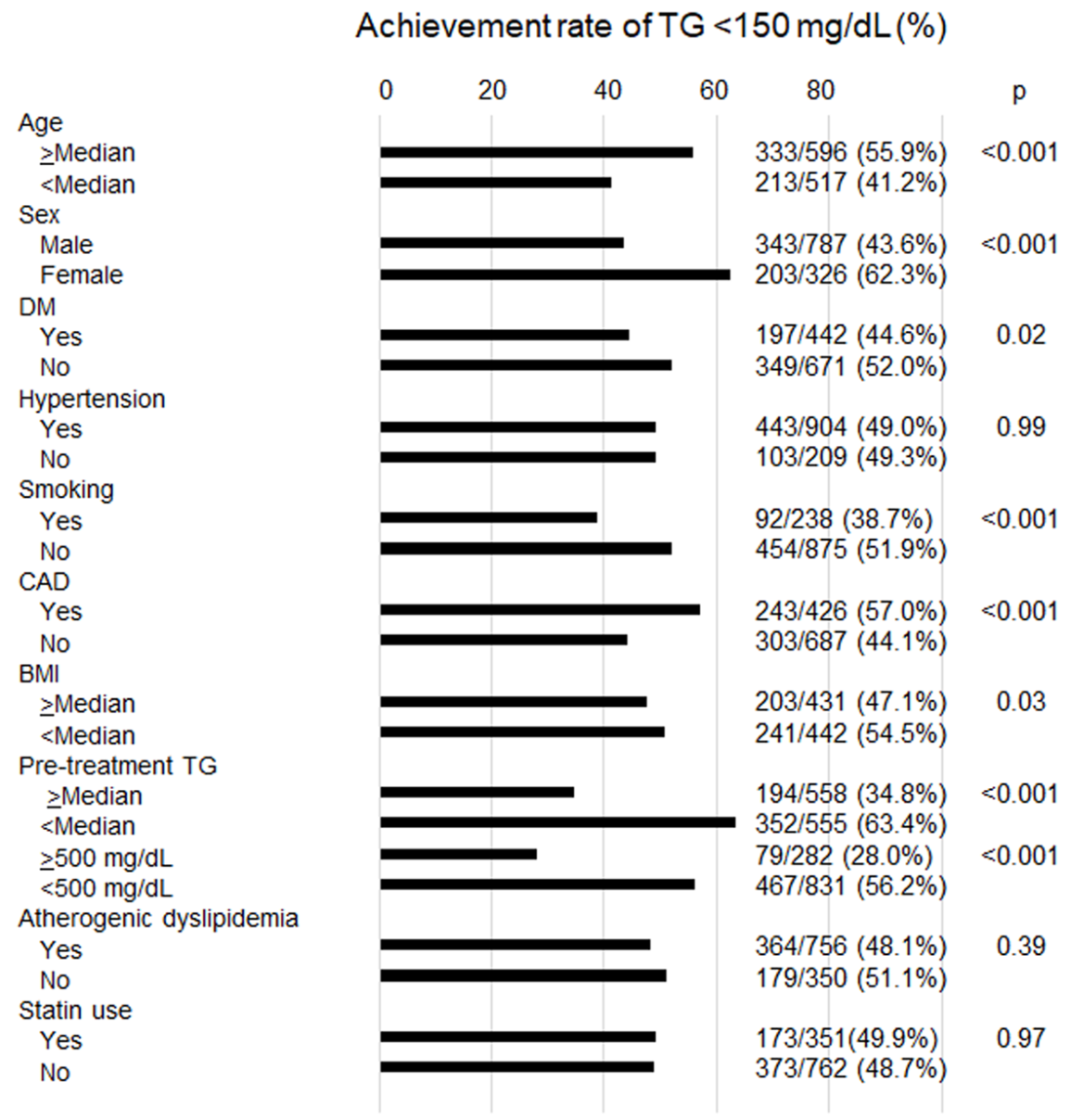


**S2 Fig. Achievement rate of TG <150 mg/dL by fenofibrate in the subgroups.** DM: diabetes mellitus; CAD: coronary artery disease; BMI: body mass index

**S1 Table. Variables associated with achievement rate of TG <150 mg/dL identified by multiple logistic regression analysis.**

| Variables | TG <150 mg/dL  (n=543) | TG ≥150 mg/dL  (n=563) | OR (95% CI) | P |
| --- | --- | --- | --- | --- |
| Age, year | 62.6 ± 11.1 | 58.4 ± 12.0 | 0.99 (0.98, 1.00) | 0.12 |
| Male | 341 (62.8) | 440 (78.2) | 2.22 (1.60, 3.08) | <0.001 |
| Diabetes mellitus | 194 (35.7) | 243 (43.2) | 1.52 (1.15, 2.01) | 0.003 |
| Hypertension | 440 (81.0) | 449 (81.5) | 1.28 (0.91, 1.81) | 0.15 |
| Current smoking | 92 (16.9) | 146 (25.9) | 1.29 (0.93, 1.80) | 0.13 |
| CAD | 243 (44.8) | 183 (32.5) | 0.47 (0.35, 0.64) | <0.001 |
| Pre-treatment TG | 304 (242, 400) | 427 (315, 601) | 1.003 (1.002, 1.004) | <0.001 |
| Atherogenic dyslipidemia | 364 (67.0) | 392 (69.6) | 1.09 (0.82, 1.46) | 0.56 |
| Statin use | 173 (31.9) | 178 (31.6) | 1.46 (1.08, 1.99) | 0.02 |

TG: triglyceride; OR: odds ratio; CI: confidence interval; CAD: coronary artery disease
